# Unveiling the Comorbidities of Chronic Diseases in Serbia Using ML Algorithms and Kohonen Self-Organizing Maps for Personalized Healthcare Frameworks

**DOI:** 10.3390/jpm13071032

**Published:** 2023-06-22

**Authors:** Nevena Rankovic, Dragica Rankovic, Igor Lukic, Nikola Savic, Verica Jovanovic

**Affiliations:** 1 Department of Cognitive Science and Artificial Intelligence, School of Humanities and Digital Sciences, Tilburg University, 5037 AB Tilburg, The Netherlands; 2 Department of Mathematics, Informatics and Statistics, Faculty of Applied Sciences, Union University “Nikola Tesla”, 18 000 Nis, Serbia; nr17031994@gmail.com; 3 Department of Preventive Medicine, Faculty of Medical Sciences, University of Kragujevac, 34 000 Kragujevac, Serbia; igorlukicvaljevo@gmail.com; 4 Department of Healthcare, Faculty of Business Valjevo, Singidunum University, 14 000 Valjevo, Serbia; nikolasavicvzs@gmail.com; 5 Institute of the Public Health “Dr. Milan Jovanovic Batut”, 11 000 Belgrade, Serbia; verica.jovanovic@singidunum.ac.rs

**Keywords:** chronic non–communicable diseases, comorbidities, prevalence, ML algorithms, Kohonen SOM neural network

## Abstract

In previous years, significant attempts have been made to enhance computer-aided diagnosis and prediction applications. This paper presents the results obtained using different machine learning (ML) algorithms and a special type of a neural network map to uncover previously unknown comorbidities associated with chronic diseases, allowing for fast, accurate, and precise predictions. Furthermore, we are presenting a comparative study on different artificial intelligence (AI) tools like the Kohonen self-organizing map (SOM) neural network, random forest, and decision tree for predicting 17 different chronic non-communicable diseases such as asthma, chronic lung diseases, myocardial infarction, coronary heart disease, hypertension, stroke, arthrosis, lower back diseases, cervical spine diseases, diabetes mellitus, allergies, liver cirrhosis, urinary tract diseases, kidney diseases, depression, high cholesterol, and cancer. The research was developed as an observational cross-sectional study through the support of the European Union project, with the data collected from the largest Institute of Public Health “Dr. Milan Jovanovic Batut” in Serbia. The study found that hypertension is the most prevalent disease in Sumadija and western Serbia region, affecting 9.8% of the population, and it is particularly prominent in the age group of 65 to 74 years, with a prevalence rate of 33.2%. The use of Random Forest algorithms can also aid in identifying comorbidities associated with hypertension, with the highest number of comorbidities established as 11. These findings highlight the potential for ML algorithms to provide accurate and personalized diagnoses, identify risk factors and interventions, and ultimately improve patient outcomes while reducing healthcare costs. Moreover, they will be utilized to develop targeted public health interventions and policies for future healthcare frameworks to reduce the burden of chronic diseases in Serbia.

## 1. Introduction

The potential benefits of artificial intelligence tools in medicine and healthcare have been extensively discussed in recent years. One of the biggest advantages of using these algorithms is the fact that they are capable of dealing with a large amount of data, such as different parameters, diagnoses obtained according to different risk factors, patient files, and many more points that should be considered when performing analysis [1,2]. Moreover, applying an ML algorithm, for example, will produce findings much more quickly, enabling the therapy to be initiated sooner. Another aid of applying intelligent techniques in healthcare is the partial elimination of human involvement, which lowers the risk of human mistakes [3]. According to the survey obtained in Serbia, with the support of the European Union project, one of the prerequisites for evaluating the population’s overall health is keeping an eye on the prevalence of chronic non-communicable diseases [4,5]. Often-used, typical statistical methods for assessment of disease development are lengthy due to the processing and analysis of all critical parameters in addition to the time required for other investigations [6].

The random forest machine learning algorithm is a supervised algorithm used for classifying or regressing data. This algorithm is useful for identifying the presence or absence of chronic diseases based on a dataset, such as individual health data and diagnostic data. Using the random forest algorithm, we can build a model that predicts the presence or absence of specific chronic diseases such as hypertension, diabetes, heart disease, and others [7,8]. Additionally, the algorithm allows us to identify key variables that have the most significant impact on the development of a particular disease, such as age, gender, body mass index, blood pressure, and other factors. Using the random forest algorithm for data analysis enables us to identify causes and risk factors for various chronic diseases. Based on this knowledge, healthcare professionals can develop prevention, diagnosis, and treatment strategies for individuals at a higher risk for developing these diseases. This is an important tool in the fight against chronic diseases and improving the quality of life for patients.

The decision tree machine learning algorithm works by creating a decision tree, where data are split into groups using different variables and rules applied to the data [9,10]. Based on these variables and rules, the model can predict the presence or absence of chronic diseases. When using the decision tree algorithm, the model is trained on a dataset containing information about individual health and diagnostic data, such as blood pressure, blood sugar levels, cholesterol levels, etc. These data are used to split the data into groups and create a decision tree used to predict the presence or absence of specific chronic diseases. The decision tree algorithm can provide insights into which variables (such as age, gender, body mass index, blood pressure, etc.) have the most significant impact on the development of a particular disease [11,12,13]. Additionally, this algorithm can be used to create a model that can predict the presence or absence of chronic diseases based on a dataset. Using the decision tree algorithm for data analysis can help healthcare professionals identify the causes and risk factors of various chronic diseases, and develop prevention, diagnosis, and treatment strategies for individuals at a higher risk for developing these diseases.

Self-organizing maps (SOMs) are a type of neural network that can be applied to analyze a dataset to identify patterns and group similar data. This approach can also be used to recognize the presence or absence of chronic diseases [14,15]. When applying the SOM neural network, the dataset is first processed to reduce the number of variables, making analysis easier. Afterward, the data are organized into a 2D map using self-organizing algorithms. This map displays data clustering by similarity and enables the identification of patterns within the data. Using the SOM neural network can aid in identifying patterns and data grouping that indicate the presence of chronic diseases. Additionally, this approach can be used to recognize complex patterns among variables that may indicate a relationship between chronic diseases and other variables such as age, gender, body mass index, blood pressure, blood sugar levels, etc. Applying the SOM neural network to a dataset allows for an insight into which variables impact the development of a certain disease the most and how these variables are interrelated. Furthermore, this approach can be used to create a model that predicts the presence or absence of chronic diseases based on a dataset [14,16].

ML algorithms and SOM neural networks can be successfully used for data analysis and can help healthcare professionals identify the causes and risk factors of various chronic diseases, and develop prevention, diagnostic, and treatment strategies for individuals who are at higher risk for developing these diseases.

In this paper, we will identify the most prevalent chronic diseases among the population of Serbia by region and age group, as well as its comorbidities. Furthermore, this study, attempts to contribute to society and science by being the first to compare the most common and popular ML algorithms and SOM neural networks to identify how 17 chronic non-communicable diseases are spread among the regions and different age groups of the residents in Serbia. Moreover, the presence of comorbidity was examined using the random forest algorithm for the most prevalent diseases.

The rest of the paper is organized as follows: Section 2 gives an overview of state-of-the-art papers discussing improvements in medical diagnostics using statistical methodologies, ML algorithms, and neural networks. Section 3 describes the methodology. Section 4 presents the obtained results. In Section 5, a discussion of the obtained results is presented, while in Section 6, concluding remarks are given.

## 2. Related Work

Medical diagnostics and general prevention using newly developed AI technologies as predictive models are rapidly growing dynamic fields of research. The results in [4] showed the effectiveness of the classification techniques of patient data, diabetes, heart disease, and cancer. Another study [5] presents the Random Forest classifier, along with chi-square (χ^2^) test and mutual information (MI) procedures to predict cancer biopsy outcomes from BI-RAD findings and the patients’ age. The authors in [6] used random forest to improve the linear discriminant analysis in order to predict the abnormal regulation of long non-coding RNA (lncRNA). For real-time healthcare analytic applications, one study [7] proposed pruned Random Forest for detecting three different diseases using different medical datasets. The study described in [8] proposed a prediction model for the classification of diabetes mellitus using the synthetic minority oversampling technique, genetic algorithm, and decision tree, with an accuracy of 82.12%. In [9], the authors compared the accuracy of the decision tree with other classification algorithms to examine a thyroid disease dataset. Another study [10] showed a comparative analysis of different ML algorithms to automate diagnosis in the medical imaging field, where the decision tree performed second best. Research presented in [11] aimed to construct the Kohonen self-organizing maps to explore the structural differences between the temperatures of patients with positive and negative oncological diagnoses of breast cancer. Another case study on thyroid biopsies [12,17] presented an SOM neural network as an alternative approach for identifying hidden patterns in biomedical data. The study [13,18] aimed to develop a predictive method for heart disease diagnosis using machine learning techniques such as principal component analysis (PCA), self-organizing map, and fuzzy support vector machine (fuzzy SVM), reducing the computation time of disease diagnosis. 

The authors of reference [19] conducted research on various comorbidities or related diseases that often occur together with hypertension, as well as their mechanisms and consequences. The authors stated that hypertension can be associated with various cardiovascular diseases, kidney disease, diabetes, stroke, depression and anxiety, obesity, respiratory diseases and vision problems. The review emphasizes the importance of regular blood pressure monitoring and the treatment of related health problems to reduce the risk of serious complications in people with hypertension. In [20], the authors focus on the interrelationship between hypertension and diabetes mellitus, two of the most common health problems in the world. The paper describes the pathophysiology of both conditions, their risk factors, and complications. The paper “Hypertension and kidney disease: A deadly connection” describes the link between hypertension and kidney disease. The authors of the research study in reference [21] point out that hypertension is one of the leading causes of kidney damage and kidney disease worldwide. The paper focuses on the causes and mechanisms that lead to kidney damage due to hypertension, as well as on the treatment of hypertension in people with kidney disease. The paper explains how an elevated blood pressure can lead to blood vessel damage in the kidneys, which can result in a reduced kidney function. The authors emphasize that an early diagnosis of hypertension and blood pressure control are crucial in preventing kidney damage. The paper by the authors of [22] describes the relationship between hypertension and recurrent stroke, analyzing different types of ischemic stroke. The study showed that hypertension is a significant risk factor for recurrent ischemic stroke, but that this association depends on the subtype of ischemic stroke. The paper describes different categories of ischemic stroke, such as large artery stroke, small artery stroke, and cryptogenic stroke, and states that hypertension is particularly associated with the recurrence of large artery strokes [23]. The paper also describes the mechanisms that explain the link between hypertension and recurrent stroke, including damage to blood vessels, atherosclerosis, increased heart volume, and other organic changes. These mechanisms emphasize the importance of hypertension control in preventing recurrent ischemic stroke. The paper states that hypertension and diabetes mellitus often occur together, significantly increasing the risk of cardiovascular and kidney diseases. The paper describes how hypertension and diabetes mellitus cause damage to blood vessels and how this leads to an increased risk of heart attack, stroke, kidney failure, and other complications. The research study presented in reference [24] describes the link between hypertension, and depression and anxiety disorders in adults in South Korea. The study was based on data obtained from a national survey conducted in South Korea, in which more than 20,000 adults participated.

In the world population, as well as in Serbia, cardiovascular diseases have been the most prevalent group of diseases for many years, with hypertension being the most common. Hypertension, or high blood pressure, is a condition characterized by high pressure in the arteries. Primary (essential) hypertension is the most common and often appears without a recognizable cause, while secondary hypertension occurs due to other health conditions. Along with hypertension, other conditions or diseases often occur, known as comorbidities. Cardiovascular disease, kidney disease, diabetes, stroke, depression and anxiety, overweight, respiratory diseases, as well as vision problems are some of the most common comorbidities associated with hypertension. Controlling blood pressure and monitoring other health problems are crucial in reducing the risk of serious complications associated with these comorbidities [25,26].

Despite the increasing number of publications in this field, to the best of the authors’ knowledge, we could not find observational cross-sectional studies exanimating 17 most common chronic non-communicable diseases simultaneously, using ML algorithms and SOM neural networks on real datasets (in this case collected from the largest Institute of Public Health “Dr. Milan Jovanovic Batut” in Serbia). 

## 3. Methodology

In order to achieve the main research goals, in Section 3.1, we will present the AI tools chosen in this research, such as decision tree and random forest, and Kohonen self-organizing map (SOM) neural network. In Section 3.2, the description of the dataset used will be given.

### 3.1. Decision Tree, Random Forest, and SOM Neural Network

The reason for utilizing decision trees is to create a training model that can be used to anticipate the class or worth of an objective variable by gaining basic decision principles obtained from past (training) data. Record class labels can be predicted using decision trees that begin at the tree’s root. Since decision trees belong to a supervised approach, pre-processed data are used to feed the algorithm. The algorithm is trained with these data. The method is top–down, with the root node always located at the top of the structure and the tree leaves acting as placeholders for the results. The main idea is to use a decision tree to divide the data space into dense and sparse areas. A decision tree that can be used to produce the best-sorted expectations is returned at the end of the training phase. This algorithm is commonly used in medical diagnostics, where it can help doctors and medical professionals make more accurate and timely diagnoses. In medical assessment, the input variables might include symptoms, patient history, and lab test results. By analyzing these data, decision trees can help identify potential diseases or conditions, and recommend appropriate treatment options. The use of decision trees in medical diagnostics can help improve the accuracy and speed of diagnoses, leading to better patient outcomes and more efficient healthcare delivery. In this research, the parameters that will be used are the max depth, min samples split, min samples leaf, max features, and min impurity decrease [27,28]. Moreover, there are different algorithms and formulas used to build a decision tree for medical diagnostics, and the choice of algorithm will depend on the specific data and problem at hand. However, one common formula used to evaluate the quality of a split in a decision tree is the Gini impurity or entropy. The Gini impurity measures the probability of incorrectly classifying a randomly chosen element in the dataset, while entropy measures the level of disorder or uncertainty in the dataset. The formula for calculating the Gini impurity is (1):

(1) $$Gini\;impurity=1-\sum {\left(pi\right)}^{2}$$

where *pi* is the probability of the occurrence of a certain class or outcome. The formula for calculating the entropy is (2):

(2) $$Entropy=-\sum pi\times \left(pi\right)$$

where *pi* is the probability of the occurrence of a certain class or outcome. These formulas are used to determine the optimal splits in the decision tree based on the input variables and outcomes of interest.

Random forest is a popular machine learning technique that has been used in medical diagnostics to improve the accuracy and robustness of diagnostic models. Random forest is a type of ensemble learning algorithm that builds multiple decision trees using different subsets of the data and input variables. Each decision tree is trained on a random subset of the data and a random subset of input variables, and the final output of the random forest is the aggregate prediction of all the trees. This approach helps reduce overfitting and increases the stability and generalizability of the model. In medical evaluation, random forest can be used to analyze a wide range of patient data, including medical history, symptoms, and imaging results, to identify potential diseases or conditions. Random forest can also be used to prioritize diagnostic tests, identify subgroups of patients that may require specialized treatment, and monitor the effectiveness of treatment over time. The ability of random forest to handle large and complex datasets, and its ability to handle missing data and noise, make it a valuable tool for medical diagnostics. Random forest is an algorithm that is trained through bagging or bootstrap aggregating. Bagging is a meta-algorithm that improves the exactness of machine learning algorithms. As the name suggests, it has a large number of individual decision trees that operate as an ensemble. The lack of correlation between the models is the key. Similar to how assets with low correlations combine to form a portfolio that is greater than the sum of its parts, uncorrelated models can provide ensemble forecasts that are more accurate than any individual predictions. The trees protect one another from their own mistakes as long as they do not always make the same mistake in the same direction. The group of trees will be able to move in the right direction because many of them will be correct and some may be wrong. In addition, random forest provides many parameters that can be adjusted. In this study, the following parameters will be the primary focus: n estimators, max features, max depth, and criterion [29,30]. 

The formula used to aggregate the outputs of multiple decision trees is the out-of-bag (OOB) error, which is used to estimate the generalization error of the random forest model. The OOB error estimate is calculated by running each data point in the training set through the decision trees that were not trained on that data point, and then aggregating the outputs of those decision trees to make a prediction. The difference between the predicted value and the true value is then used to estimate the OOB error (3). Another formula used in random forest is the variable importance measure, which is used to determine the importance of each input variable in the model. The variable importance measure is calculated by comparing the accuracy of the random forest model with and without each input variable. The difference in accuracy is then used to estimate the importance of each variable in the model. The variable importance measure can help identify the most informative input variables and can be used to refine the model and improve its accuracy.

(3) OOB error estimate=1n×∑yi−yi^2

where *n* is the number of data points in the training set, *y_i_* is the true value of the ith data point, and *ŷ_i_* is the predicted value of the ith data point using only the decision trees that did not use that data point in their training. The *OOB error estimate* is calculated by running each data point in the training set through the decision trees that were not trained on that data point, and then aggregating the outputs of those decision trees to make a prediction. The difference between the predicted value and the true value is then squared and summed over all data points in the training set. The *OOB error estimate* provides an unbiased estimate of the generalization error of the random forest model, as it is calculated using data that were not used in the training of the decision trees [30].

Among the fundamental types of self-organizing neural networks are Kohonen’s networks. Kohonen self-organizing maps (SOMs) have been used in medical diagnostics to identify patterns and relationships within complex patient data. SOM works by mapping high-dimensional data onto a low-dimensional grid, where each cell on the grid represents a specific feature or pattern in the data. An SOM can be trained on large and complex datasets, including medical imaging data, patient history, and genetic data, to identify the subgroups of patients that share similar characteristics, or to identify features that are associated with specific diseases or conditions. In medical assessment, SOM has been used to identify patterns in medical imaging data, such as identifying tumors or abnormal tissue growth. SOMs have also been used to classify patients based on their symptoms and medical history, and identify the subgroups of patients that may require specialized treatment. The ability of SOMs to handle large and complex datasets, and identify relationships and patterns that may be difficult to detect using traditional statistical methods, makes it a valuable tool for medical diagnostics [31,32]. 

The capacity to self-organize opens up new possibilities, such as adapting to input data that were previously unknown. It seems to be the most natural way to learn because these networks create a group of networks that employs a competitive, self-organizing learning strategy. The precise strategy of competition and subsequent adjustments to synaptic weights may take a variety of forms. There are numerous rivalry-based subtypes that distinguish themselves through precise self-organizing algorithms. Regarding the structure, the most common type of network architecture is one-way, one-layer. The requirement establishes that all neurons participate in the competition with equal rights. As a result, each one needs to have as many inputs as the entire system. The weighting rule is given as follows (4), (5):

(4) $$w_{ij}\left(t+1\right)=w_{ij}\left(t\right)+\alpha_{i}\left(t\right)\cdot \left(x\left(t\right)-w_{ij}\left(t\right)\right),\;$$

(5) $$w_{ij}\left(t+1\right)=w_{ij}\left(t\right)+\alpha_{i}\left(t\right)\cdot \beta_{i}\left(t\right)\left(x\left(t\right)-w_{ij}\left(t\right)\right)$$

where *α* is a learning rate at time *t*, *j* denotes the winning vector, *i* denotes the *i^th^* feature of the training example. Trained weights are utilized for clustering new examples, where a new example is in the cluster of winning vectors [31,33]. 

Over time, both the radius and learning rate undergo exponential decay that is similar in nature, along with the neighborhood function influence $\beta_{i}$(*t*) (6), (7), (8).

(6) $$\sigma \left(t\right)=\sigma \cdot exp\left(\frac{-t}{\lambda }\right),$$

(7) $$\sigma \left(t\right)=\alpha_{0}\cdot exp\left(\frac{-t}{\lambda }\right),$$

(8) $$\beta_{ij}\left(t\right)=exp\left(\frac{-d^{2}}{2\sigma^{2}\left(t\right)}\right),\;\mathrm{where}\;t=1,\;2,\;3,\;\ldots \;n.$$

The best matching unit (BMU) is selected from the smallest calculated node distances, according to the following Formula (9):

(9) $$d=\min\left(\left\|\vec{x}-\vec{w_{ij}}\right\|\right)=min\left(\sqrt{\sum_{t=0}^{n}{\left[\vec{x\left(t\right)}-\vec{w_{ij}}\left(t\right)\right]}^{2}}\right)$$

### 3.2. Dataset Description

The presented research started in 2019 as an observational cross-sectional study. It includes 13,178 respondents (residents) from all over Serbia. There were 6431 (48.8%) male and 6747 (51.2%) female respondents. Respondents from all over Serbia come from four different regions. There were 3061 (23.2%) respondents from Belgrade, 2963 (22.5%) from Vojvodina, 4233 (32.1%) from Sumadija and western Serbia, and 2921 (22.2%) from eastern and southern Serbia. The age groups of the respondents included 15 to 24 years (*n* = 1519, 11.5%), 25 to 34 years (*n* = 1629, 12.4%), 35 to 44 years (*n* = 1949, 14.8%), 45 to 54 years (*n* = 1989, 15.1%), 55 to 64 years (*n* = 2387, 18.1%), 65 to 74 years (*n* = 2297, 17.4%), 75 to 84 years (*n* = 1125, 8.5%), and over 85 years old (*n* = 283, 8.5%). Considering that this is a national study, there was a minimal number of missing data in the proposed research, which was ignored in the analysis. Furthermore, the experiment was performed on data that were highly unbalanced. Additionally, the unbalanced dataset was a significant issue in medical diagnostics; in this case, the clustering method was used to convert the data into balanced data in the pre-processing phase. 

## 4. Results

In this section, we will present the main results obtained using traditional statistical techniques, ML algorithms, and SOM neural networks. Table 1, Table 2 and Table 3 present the percentage share of each of the 17 observed chronic non-communicable diseases by region and age group. It can be concluded that the most significant percentage of residents suffer from cardiovascular diseases, and the most common one from that group is hypertension, regardless of the region and age group. Moreover, the presence of non-communicable diseases is higher in underdeveloped regions such as the region of Sumadija and western Serbia and the region of southern and eastern Serbia compared to more developed regions of Belgrade and Vojvodina. The spread of all diseases was most prevalent in the age range of 65 to 74 years. The frequency of the spread of almost all diseases was significantly higher in females compared to males. The incidence rate of hypertension represented 31.1% of all the chronic non-communicable diseases studied.

**Figure 1:**
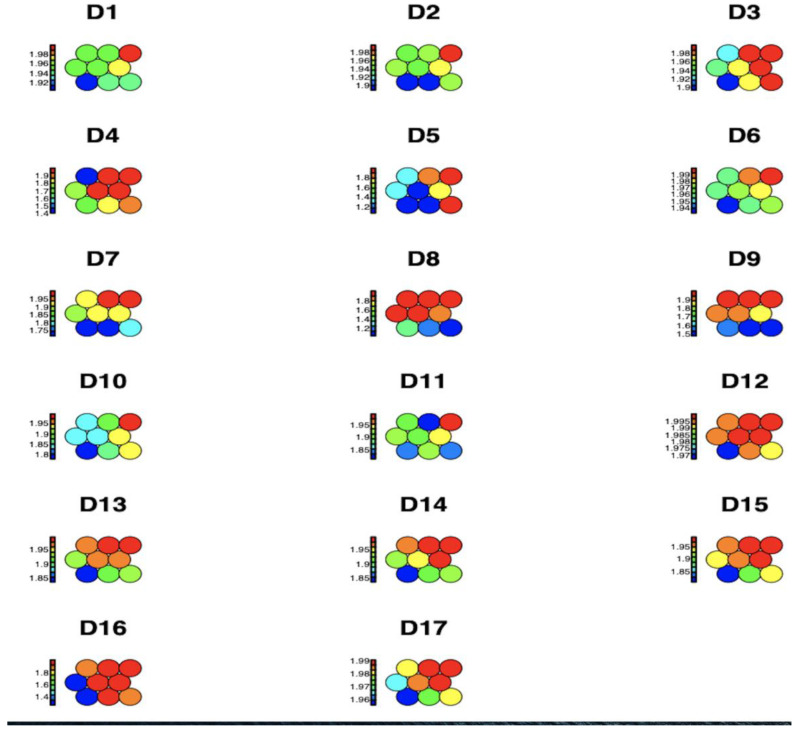
Graphical representation of the prevalence of diseases per region and age group using a Kohonen SOM neural network.

Figure 1 shows the average impact values of each of the 17 observed chronic non-communicable diseases by region and age group within each node, represented through a heat map for a Kohonen SOM neural network. The higher disease concentrations are shown in red, while medium and lower disease spread concentrations are shown in blue, green, and yellow, respectively. It can be concluded that D16 High_Cholesterol is identified as having the highest prevalence.

**Figure 2:**
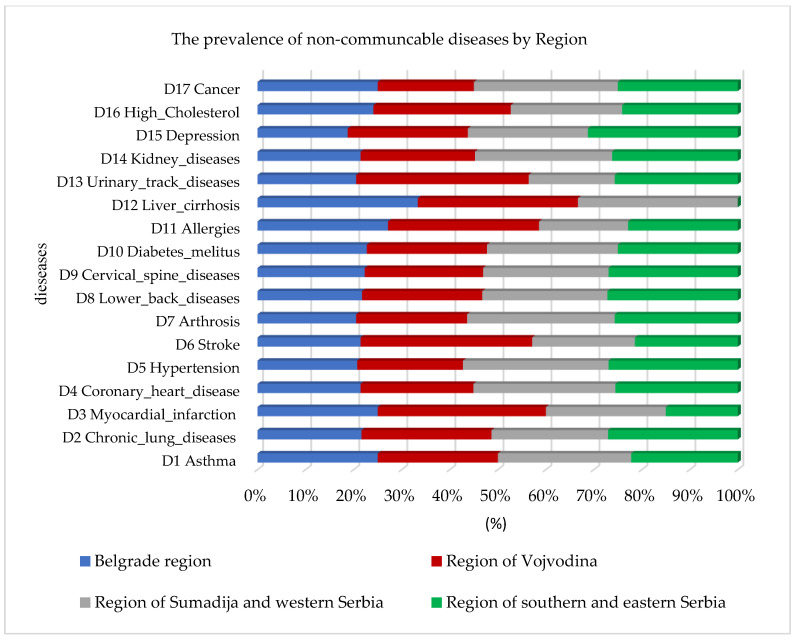
Graphical representation of the prevalence of non-communicable diseases by region using random forest.

In addition to the prevalence of cardiovascular diseases, the random forest algorithm also identified a higher prevalence of high cholesterol and diabetes mellitus, as well as lung diseases and asthma, observed by region in Figure 2. The highest percentage of prevalence of all chronic non-communicable diseases in comparison to the other regions was identified in the Sumadija and western Serbia region, with a contribution of 32.1%.

**Figure 3:**
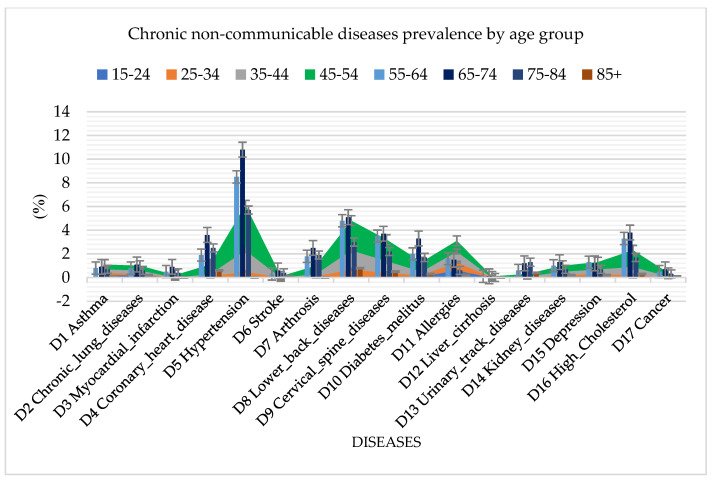
Graphical representation of chronic non–communicable disease prevalence by age group using random forest.

Analyzing the spread of diseases by age groups, in addition to cardiovascular diseases, which are the common diseases among the population in Serbia, the random forest algorithm also identified a high presence of diabetes mellitus, arthrosis, urinary tract diseases, and high cholesterol (Figure 3). The highest incidence rate of all diseases was found in the age group of 65 to 74 years, which reached a percentage of 42.3%.

**Figure 4:**
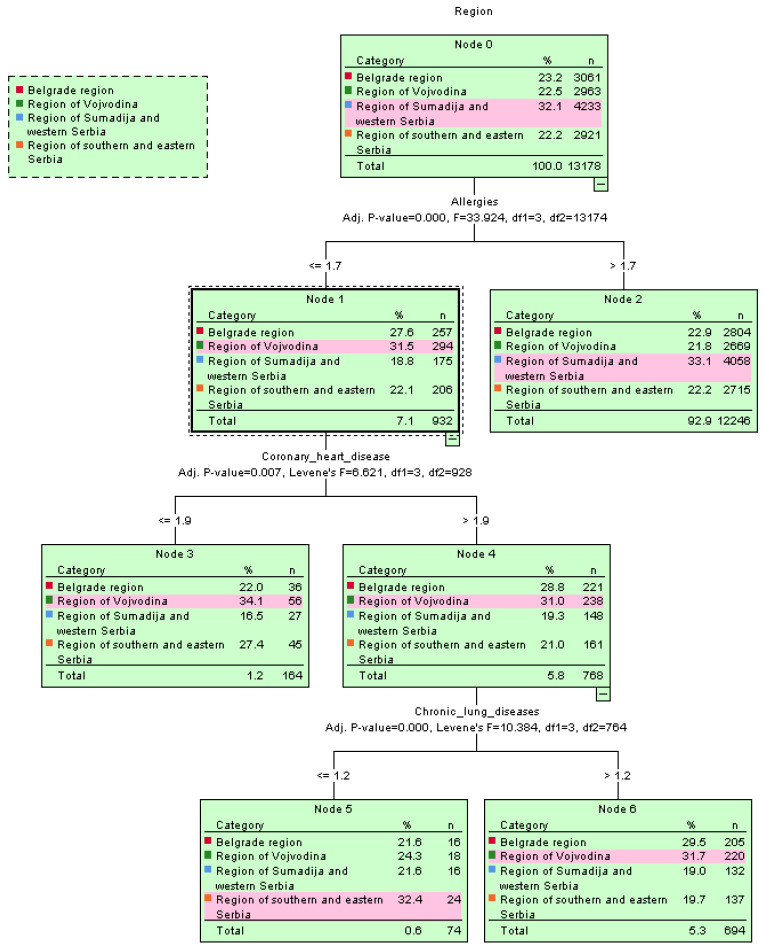
Graphical representation of the spread of diseases per region using the decision tree classifier.

The spread of cardiovascular diseases with hypertension as a common health issue among the residents of Serbia was confirmed using the decision tree algorithm, shown in the tree’s root structure, e.g., by region in Figure 4.

**Figure 5:**
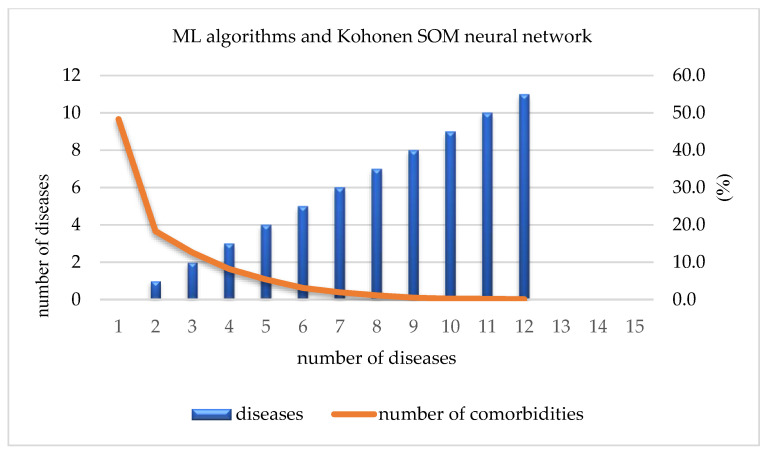
Number of comorbidities.

By using ML algorithms and a Kohonen SOM neural network, it is possible to define the number and percentage of present diseases in the overall sample. Out of a total of 13,178 participants, 6365 (48.3%) did not have any of the 17 investigated chronic non-communicable diseases. The number of participants that were affected by only one chronic non-communicable disease reached 2413 (18.3%), while that of those affected by two diseases reached 1663 (12.6%). This percentage was reduced to only 10% for participants suffering from three or more chronic non-communicable diseases. On the other hand, only 2 out 13,178 participants were suffering from 13 out of 17 chronic non-communicable diseases (Table 3, Figure 5).

Hypertension, also known as high blood pressure, can be associated with various other health problems, referred to as comorbidities. Some of the most common comorbidities of hypertension include diabetes, cardiovascular diseases, obesity, kidney diseases, hyperlipidemia, anxiety, and depression. High blood pressure and diabetes are often linked, as the causes of one can be associated with the causes of the other. Additionally, diabetes can exacerbate hypertension. This disease is a significant risk factor for the development of heart diseases such as heart attack, angina, and stroke. Obesity and hypertension are also closely related, as overweight can increase the risk of hypertension, and hypertension can worsen obesity. These two conditions together increase the risk of other comorbidities. Hypertension can also damage the blood vessels in the kidneys, leading to various kidney diseases, including chronic kidney disease. Increased concentrations of fats in the blood, such as cholesterol and triglycerides, can also increase the risk of hypertension and heart diseases. Finally, individuals with hypertension often suffer from anxiety and depression. These psychological conditions can worsen hypertension and make successful treatment more difficult.

**Figure 6:**
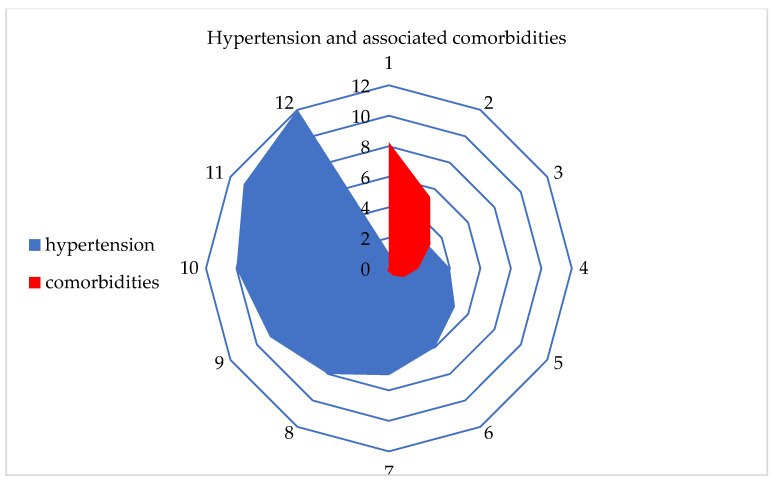
Graphical representation of hypertension and the associated comorbidities.

After using various ML algorithms and a Kohonen SOM neural network, hypertension was identified as the most influential chronic non-communicable disease among residents in Serbia. Additionally, the number and percentage of comorbidities associated with hypertension were examined. It was found that at least 1 comorbidity was present in 8.3% of the participants, while the highest number of comorbidities, which was 11, was found in only two respondents (Table 4, Figure 6).

Similar to chronic non-communicable diseases, machine learning algorithms have identified comorbidities of hypertension prevalent by region. The highest percentage of comorbidity of hypertension was found in the Sumadija and western Serbia region at 8.2%. The Vojvodina region had 4.8% comorbidity of hypertension, followed by the Belgrade region with 4.3%. The lowest percentage (0.3%) of comorbidity was found in the eastern and southern Serbia regions.

**Figure 7:**
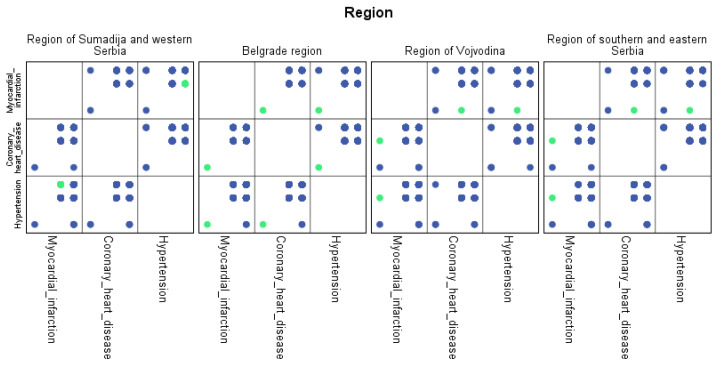
Graphical representation of comorbidities of hypertension and associated comorbidities by region.

**Figure 8:**
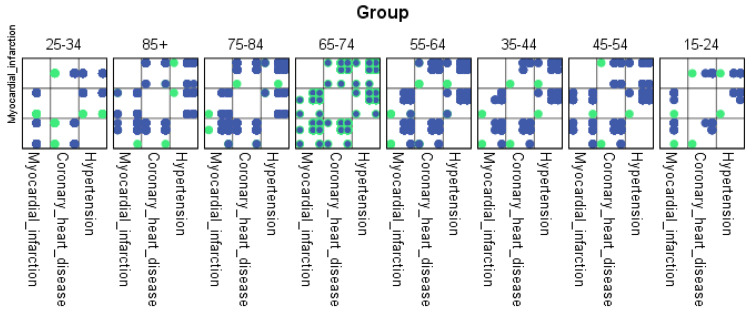
Graphical representation of comorbidities of hypertension and associated comorbidities by age group.

**Figure 9:**
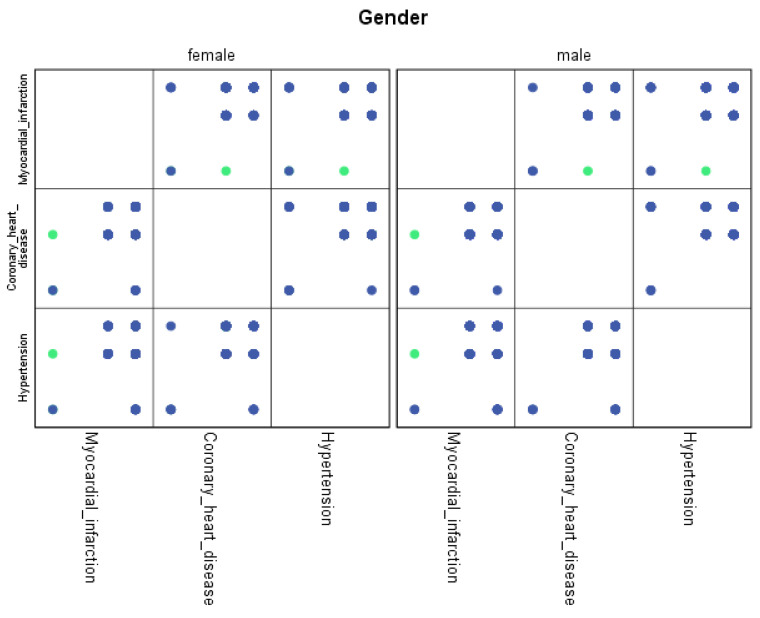
Graphical representation of comorbidities of hypertension and associated comorbidities by gender.

For each region, machine learning algorithms have identified the presence of one or more comorbidities, up to a maximum of six. The Belgrade region displayed the presence of one comorbidity at 1.9%, two comorbidities at 1.1%, three comorbidities at 0.7%, four at 0.4%, and the presence of five and six comorbidities at 0.1%. The Vojvodina region showed the presence of one comorbidity at 2.1%, two comorbidities at 1.2%, three comorbidities at 0.6%, four at 0.5%, and the presence of five at 0.3% and six comorbidities at 0.1%. The Sumadija and western Serbia region revealed the presence of one comorbidity at 3.2%, two comorbidities at 2.2%, three comorbidities at 1.3%, four at 0.7%, and the presence of five at 0.6% and six comorbidities at 0.2%. The southern and eastern Serbia regions showed the presence of one comorbidity at 1.1%, two comorbidities at 0.9%, three comorbidities at 0.5%, four at 0.3%, and the presence of five and six comorbidities at 0.1%. Graphical representations of the obtained results manifesting the hypertension as a leading (a common) disease with associated comorbidities have been shown in Figure 7, by region, in Figure 8 by group, and in Figure 9 by gender. In Figure 7, Figure 8 and Figure 9, the blue dots represent the highest prevalence of comorbidities in relation to the three most influential cardiovascular diseases: hypertension, coronary heart disease, and myocardial infarction. The green dots represent the lowest presence of comorbidities for the aforementioned three diseases by region, age group and gender, respectively.

## 5. Discussion

The societal and scientific contribution of this study can be seen through its main goals. The first goal was to identify the prevalence of 17 chronic non-communicable diseases by region and age group among the residents of Serbia. Based on the results obtained in Section 4, it can be concluded that the spread of chronic non-communicable diseases is higher in less developed regions of Serbia such as Sumadija and western Serbia, with average values of 2.1%, or 277 residents. On the other hand, all diseases are the least present in the most developed region of Serbia, the Belgrade region, with an average of 1.7%, or 224 residents. 

Comparing the lowest possible error rate between the AI tools used, random forest outperformed others, with an accuracy of 1.1%, while decision tree and an SOM neural network had the values of 1.4% and 2.7, respectively. 

The spread of chronic non-communicable diseases was highest in the age group of 65 to 74 years (with an average of 10.8%, or 1423 residents). It was found that the lowest spread of non-communicable disease is found in the age group from 15 to 24 years (with an average of 0.1%, or 13 residents). It is interesting to point out that the spread of the observed diseases was significantly lower in the age group over 85 years (with an average of 0.1%, or 10 residents). Comparing the lowest possible error rate between the AI tools used, random forest outperformed the others, with an accuracy of 1.1%, while decision tree and an SOM neural network had the values of 1.8% and 3.1, respectively. Additionally, it is important to point out that the spread of most diseases is significantly higher in female than male residents by region and age group. 

The second goal was to identify the number of associated comorbidities with the leading disease, i.e., hypertension. It was found that about half of the participants did not have any of the 17 examined chronic non-communicable diseases (48.3%). Only one disease was present in 18.3% of the participants, while two diseases were present in 12.6%. Three or more present diseases were found in less than 10% of the participants. Thirteen chronic non-communicable diseases were present in only two participants.

Using machine learning algorithms and a Kohonen SOM neural network, it was determined that hypertension is the most prevalent disease among the population of Serbia, affecting about 1/3 of the total population. Hypertension has the presence of one comorbidity in 8.3% of the population, while the presence of the highest number of comorbidities of the 11 other diseases was found in two individuals.

Machine learning algorithms have identified hypertension comorbidities distributed across the region. The highest percentage (8.2%) of the comorbidity of hypertension was in the Sumadija and western Serbia region, distributed differently according to the total number of comorbidities. The Vojvodina region had 4.8% of hypertension comorbidities, followed by the Belgrade region with 4.3%. The smallest percentage (3.0%) of comorbidities was found in the eastern and southern Serbia region, distributed proportionally according to the number of present comorbidities.

In comparison to the research conducted in 2013, a marginal reduction in the prevalence of chronic non-communicable diseases has been observed in the present study. Undoubtedly, cardiovascular diseases remain the primary cause of mortality, with hypertension playing a pivotal role in this regard. Notably, the national study conducted in 2013 reported a prevalence rate of 34.0% for hypertension, whereas in the current investigation, after a span of six years of comprehensive national data collection, the prevalence of this condition stands at 32.3% [34].

Interestingly, a majority of other diseases exhibited a decline of approximately 1% in their prevalence, indicating potential positive trends in disease management and public health interventions. However, it is worth noting that diabetes mellitus stands as an exception, exhibiting a slight increase of 0.3% in its prevalence over the same period. This finding warrants further investigation and highlights the need for targeted strategies to address the rising burden of diabetes in the population [35].

These findings underscore the importance of ongoing surveillance and research efforts to monitor the trends in chronic non-communicable diseases and identify effective interventions. The observed reduction in the prevalence of most diseases signifies progress in public health initiatives and underscores the importance of multifaceted approaches encompassing lifestyle modifications, early detection, and evidence-based management strategies. Nonetheless, continued efforts are warranted to further mitigate the impact of these diseases and implement preventive measures that can contribute to improved health outcomes and reduce mortality rates in the population.

### 5.1. Limitations

When utilizing Kohonen self-organizing maps for large-scale medical datasets, several considerations should be taken into account. Training SOM neural networks on such datasets can be computationally demanding and time-consuming, as demonstrated in this study, which involved a dataset comprising 13,178 instances with more than 20 relevant features. Proper preprocessing of the data and optimization of SOM parameters are crucial steps in this study. Additionally, while SOMs provide valuable insights into the overall structure of the data, they may not uncover hidden concepts associated with indirect biases in the data, posing a challenge in result interpretation. Moreover, achieving convergence to an optimal solution is not always guaranteed, necessitating the conduction of multiple experiments to validate the final outcomes. To address this, experiments with different parameter values, such as gradually decreasing the learning rate or utilizing adaptive learning rate schedules can enhance convergence, and were checked in this study accordingly. Similarly, when applying random forest and decision trees to medical datasets, certain factors should be considered. These algorithms are prone to overfitting and can be sensitive to minor changes in the training data. Additionally, interpreting the outcomes of these models in the medical domain can be intricate due to the generation of complex and often non-linear decision boundaries.

### 5.2. Future Research

In the future, research will be dedicated to analyzing the risk factors associated with the development of chronic non-communicable diseases using deep learning models. By leveraging the power of deep learning algorithms, researchers can explore vast amounts of data from diverse sources, such as electronic health records, genomics, lifestyle factors, and environmental exposures, to gain deeper insights into the multifaceted nature of chronic diseases. Therefore, the research endeavor aims to enhance our understanding of the risk factors underlying chronic non-communicable diseases and pave the way for more effective prevention, early diagnosis, and targeted interventions in the future.

## 6. Conclusions

With the rise of big data, healthcare professionals have access to vast amounts of information, which can be overwhelming to process and analyze using traditional statistical methods. ML algorithms and Kohonen SOM neural networks offer a solution to this problem by allowing for the automated analysis of large amounts of data and the identification of complex patterns that may not be immediately apparent using traditional methods. One of the key advantages of these AI tools is their ability to learn from data and improve their predictions over time. By training these algorithms on large datasets of patient health records, for example, healthcare professionals can develop highly accurate models for predicting the likelihood of chronic non-communicable diseases and their associated comorbidities in individual patients. These models can then be used to develop personalized treatment plans and interventions that are tailored to each patient’s specific needs, resulting in better health outcomes and improved quality of life. Overall, the use of ML algorithms and neural networks has the potential to revolutionize the way we approach healthcare by enabling more accurate predictions and more effective interventions for chronic non-communicable diseases and their associated comorbidities.

The results of this study can significantly contribute to surveying the prevalence of chronic non-communicable diseases among the residents of Serbia. By using the proposed AI tools, it is possible to adjust the work of the health system to improve and have a preventive effect on the entire population’s health.

## Figures and Tables

**Table 1:** Chronic non-communicable disease prevalence within different regions.

Region				
Chronic Non-Communicable Disease	Belgrade Region (%)	Region of Vojvodina (%)	Region of Sumadija and Western Serbia (%)	Region of Southern and Eastern Serbia (%)
D1 Asthma	0.9	0.9	1	0.8
D2 Chronic_lung_diseases	0.8	1	0.9	1
D3 Myocardial_infarction	0.5	0.7	0.5	0.3
D4 Coronary_heart_disease	2.1	2.3	2.9	2.5
D5 Hypertension	6.7	7.1	9.8	8.7
D6 Stroke	0.3	0.5	0.3	0.3
D7 Arthrosis	1.6	1.8	2.4	2
D8 Lower_back_diseases	4	4.6	4.8	5
D9 Cervical_spine_diseases	2.9	3.2	3.4	3.5
D10 Diabetes_melitus	2	2.2	2.4	2.2
D11 Allergies	1.9	2.2	1.3	1.6
D12 Liver_cirrhosis	0.1	0.1	0.1	0
D13 Urinary_track_diseases	0.8	1.4	0.7	1
D14 Kidney_diseases	0.9	1	1.2	1.1
D15 Depression	0.9	1.2	1.2	1.5
D16 High_Cholesterol	2.7	3.2	2.6	2.7
D17 Cancer	0.5	0.4	0.6	0.5

**Table 2:** Chronic non-communicable disease prevalence within different age groups.

Age Group								
Chronic Non-Communicable Disease	15–24 (%)	25–34 (%)	35–44 (%)	45–54 (%)	55–64 (%)	65–74 (%)	75–84 (%)	85+ (%)
D1 Asthma	0.2	0.2	0.3	0.4	0.8	0.9	0.7	0.1
D2 Chronic_lung_diseases	0.2	0.1	0.3	0.4	0.8	1.1	0.6	0.2
D3 Myocardial_infarction	0.0	0.0	0.0	0.2	0.5	0.9	0.4	0.0
D4 Coronary_heart_disease	0.0	0.1	0.2	0.8	1.9	3.6	2.5	0.6
D5 Hypertension	0.1	0.4	1.8	3.8	8.5	10.8	5.7	0.0
D6 Stroke	0.0	0.0	0.0	0.1	0.3	0.6	0.4	0.1
D7 Arthrosis	0.0	0.1	0.2	0.7	1.8	2.5	1.9	0
D8 Lower_back_diseases	0.1	0.6	1.6	2.5	4.8	5.1	3	0.8
D9 Cervical_spine_diseases	0.1	0.3	1.0	1.8	3.5	3.7	2.2	0.5
D10 Diabetes_melitus	0.1	0.1	0.3	0.8	2	3.3	1.7	0.3
D11 Allergies	0.6	0.7	0.9	0.9	1.6	1.5	0.6	0.2
D12 Liver_cirrhosis	0.0	0.0	0.0	0.1	0.1	0.1	0.0	0.0
D13 Urinary_track_diseases	0.0	0.0	0.1	0.2	0.6	1.2	1.3	0.4
D14 Kidney_diseases	0.1	0.1	0.3	0.5	1	1.3	0.7	0.2
D15 Depression	0.1	0.2	0.4	0.6	1.3	1.2	0.7	0.2
D16 High_Cholesterol	0.0	0.2	0.7	1.4	3.3	3.8	1.7	0.3
D17 Cancer	0.0	0.0	0.1	0.3	0.5	0.7	0.3	0.1

**Table 3:** ML algorithms and Kohonen SOM neural network: the number and percentage of diseases.

No. of diseases	0	1	2	3	4	5	6	7	8	9	10	11	12	13
No. of respondents	6365	2413	1663	1078	708	409	254	149	67	30	25	10	4	2
Percentage	48.3	18.3	12.6	8.2	5.4	3.1	1.9	1.1	0.5	0.2	0.2	0.1	0.0	0.0

**Table 4:** ML algorithms: the number and percentage of associated comorbidities for hypertension.

No. of comorbidities	1	2	3	4	5	6	7	8	9	10	11	12
No. of respondents	1088	711	413	254	150	69	30	25	10	4	2	0
Percentage	8.3	5.4	3.1	1.9	1.1	0.5	0.2	0.2	0.1	0.0	0.0	0.0

## Data Availability

The raw dataset used for this study is under a Non-Disclosure Agreement (NDA) and is therefore not available to the public.
